# Incidence and time trends of childhood hematological neoplasms: a 36-year population-based study in the southern European context, 1983–2018

**DOI:** 10.3389/fonc.2023.1197850

**Published:** 2023-07-25

**Authors:** Jan Trallero, Arantza Sanvisens, Fernando Almela Vich, Noura Jeghalef El Karoni, Isabel Saez Lloret, Cristina Díaz-del-Campo, Ana Isabel Marcos-Navarro, Amaia Aizpurua Atxega, Patricia Sancho Uriarte, Marta De-la-Cruz Ortega, María José Sánchez, Josefina Perucha, Paula Franch, María Dolores Chirlaque, Marcela Guevara, Alberto Ameijide, Jaume Galceran, Cristina Ramírez, Marta Rodríguez Camblor, Maria Araceli Alemán, Pilar Gutiérrez, Rafael Marcos-Gragera, REDECAN

**Affiliations:** ^1^Epidemiology Unit and Girona Cancer Registry, Oncology Coordination Plan, Catalan Institute of Oncology, Girona Biomedical Research Institute Dr. Josep Trueta (IDIBGI), Girona, Spain; ^2^Josep Carreras Leukaemia Research Institute, Badalona, Spain; ^3^Registry of Childhood and Adolescent Tumors of the Valencian Community, Valencian Community Department of Universal Health and Public Health, València, Spain; ^4^Cancer Information System of the Valencian Community, Valencian Community Department of Universal Health and Public Health, València, Spain; ^5^Castellón Cancer Registry, Directorate General of Public Health and Addictions, Valencian Government, Castellón, Spain; ^6^Ciudad Real Cancer Registry, Health and Social Welfare Authority, Ciudad Real, Spain; ^7^Cuenca Cancer Registry, Health and Social Welfare Authority, Cuenca, Spain; ^8^Basque Country Cancer Registry, Basque Government, Vitoria-Gasteiz, Spain; ^9^Consortium for Biomedical Research in Epidemiology and Public Health (CIBERESP), Madrid, Spain; ^10^Granada Cancer Registry, Andalusian School of Public Health (EASP), Instituto de Investigación Biosanitaria Ibs. GRANADA, University of Granada, Granada, Spain; ^11^Instituto de Investigación Biosanitaria Ibs.GRANADA, Granada, Spain; ^12^Department of Preventive Medicine and Public Health, University of Granada, Granada, Spain; ^13^La Rioja Cancer Registry, Epidemiology and Health Prevention Service, Logroño, Spain; ^14^Mallorca Cancer Registry, Public Health and Participation Department, Palma de Mallorca, Spain; ^15^Health Research Institute of the Balearic Islands (IdISBa), Palma de Mallorca, Spain; ^16^Department of Epidemiology, Regional Health Authority, Instituto Murciano de Investigación Biosanitaria (IMIB)-Arrixaca, Murcia University, Murcia, Spain; ^17^Navarra Cancer Registry, Navarra Public Health Institute, Pamplona, Spain; ^18^Epidemiology and Public Health Area, Navarra Institute for Health Research (IdiSNA), Pamplona, Spain; ^19^Tarragona Cancer Registry, Cancer Epidemiology and Prevention Service, Sant Joan de Reus University Hospital, Tarragona, Spain; ^20^Institut d’Investigació Sanitària Pere Virgili (IISPV), Reus, Tarragona, Spain; ^21^Albacete Cancer Registry, Health and Social Welfare Authority, Albacete, Spain; ^22^Department of Health, Asturias Cancer Registry, Public Health Directorate, Asturias, Spain; ^23^Canary Islands Cancer Registry, Public Health Directorate, Canary Islands Government, Tenerife, Spain; ^24^Castilla y León Cancer Registry, Public Health Directorate, Castilla y León Government, Valladolid, Spain; ^25^Nursing, University of Girona, Girona, Spain

**Keywords:** incidence, childhood, hematological neoplasms, incidence trends, leukemia-lymphoma, population-based registries

## Abstract

**Background:**

Hematological neoplasms (HNs) are the first and most common childhood cancers globally. Currently, there is a lack of updated population-based data on the incidence of these cancers in the Spanish pediatric population. This study aimed to describe the incidence and incidence trends of HNs in children (0–14 years) in Spain using data from the Spanish Network of Cancer Registries and to compare the results with other southern European countries.

**Methods:**

Data were extracted from 15 Spanish population-based cancer registries between 1983 and 2018. Cases were coded according to the *International Classification of Diseases for Oncology*, third edition, first revision, and grouped according to the *International Classification of Childhood Cancer*, third edition. Crude rates (CRs), age-specific rates, and age-standardized incidence rates using the 2013 European population (ASR_E_) were calculated and expressed as cases per 1,000,000 child-years. Incidence trends and annual percentage changes (APCs) were estimated.

**Results:**

A total of 4,747 HNs were recorded (59.5% boys). Age distribution [n (%)] was as follows: <1 year, 266 (5.6%); 1–4 years, 1,726 (36.4%); 5–9 years, 1,442 (30.4%); and 10–14 years, 1,313 (27.6%). Leukemias were the most common group, with a CR and an ASR_E_ of 44.0 (95%CI: 42.5; 45.5) and 44.1 (95%CI: 42.6; 45.7), respectively. The CR and ASR_E_ of lymphomas were 20.1 (95%CI: 19.1; 21.1) and 20.0 (95%CI: 19.0; 21.1), respectively. The comparable incidence rates between our results and those of other southern European countries were similar for lymphomas, while some differences were observed for leukemias. From 1988 to 2016, the trend in leukemia incidence was stable for both sexes, with an APC of 0.0 (95%CI: −0.5; 0.7), whereas a constant overall increase was observed for lymphoma in both sexes, with an APC of 1.0 (95%CI: 0.4; 1.6).

**Conclusion:**

Leukemias are the most common HNs in children, and their incidence has remained stable since 1988, whereas the incidence of lymphomas has increased every year. Lymphoma incidence is like that of other southern European countries, while leukemia incidence is similar only to that of southwestern European countries. Collaborative cancer registry projects allow for assessing epidemiological indicators for cancers such as HNs, which helps health authorities and clinicians provide more knowledge about these malignancies.

## 1 Introduction

Hematological neoplasms (HNs) are divided into leukemias and lymphomas. They account for one-third of all childhood cancers and are among the most common cancers in children (1). Leukemias are a group of diseases involving an uncontrolled proliferation of hematopoietic stem cells in the bone marrow caused by several risk factors such as genetic (e.g., chromosomal translocations, rare germline mutations, or epigenetics) and environmental factors (e.g., infections and exposure to chemicals or ionizing radiation) (2, 3). Meanwhile, lymphomas are a diverse group of diseases that arise from the clonal proliferation of lymphocytes (4). Due to the heterogeneity of these malignancies and the improvement of diagnostic methods based on genetic and pathological examinations (5), the *International Classification of Childhood Cancer* (ICCC), which classifies cancer histology codes in children, has been updated to the latest edition, the third edition (ICCC-3) of 2017 (1).

The most recent international collaborative study on childhood cancer (0–14 years), published in 2017 and covering the period 2001–2010, found an age-standardized rate (ASR) using the Segi world standard population (ASR_SEGI_) of 61.6 cases per million child-years, of which leukemias accounted for 46.4 and lymphomas for 15.2 (6). Slightly lower rates were estimated in a European study, covering the period 1988–1997, which compared childhood cancer incidence between geographical areas in Europe (7). Both studies reported higher incidence rates, particularly in southern Europe, compared with the rest of the world (6, 7).

The latest European study assessing incidence trends showed a statistically significant increase in the annual percentage change (APC) of 0.7% per year from 1991 to 2010 for childhood leukemias, whereas no increase was observed for lymphomas (8). In addition, some specific countries or regional population-based cancer registries have shown different results on childhood HN incidence trends (9, 10). Previously published data on childhood HNs in Spain covered the period 1983–2002 and reported an ASR_SEGI_ of 64.4 cases per million children, of which 49.9 cases were leukemias and 18.5 were lymphomas. The incidence trends for the same period were also discussed and showed an increase in the early years (1983–1991), followed by a stabilization in the second period (1992–2002) (11). Later studies covering a longer period up to 2013 for the incidence and up to 2007 for the incidence trends reported similar results for all HNs and leukemias (1, 12, 13); however, lymphomas showed stability throughout the period (13). The lack of updated results on the incidence and incidence trends of HNs in Spain requires an analysis of more recent years.

Previous studies have reported that developed countries tend to have higher incidence rates of HNs and southern European countries have higher incidence rates of lymphoma (6), in addition to a variation in trends (9, 10). Although stable incidence rates have previously been described in Spain (12, 13), we hypothesize that similar results to those reported in Europe will currently be observed, with higher incidence rates and changes in trends for both HN groups. Therefore, this study aims to provide an overview of the incidence and incidence trends of childhood HNs in Spain. This will be performed by adding more recent years and coverage areas to the previous studies, taking into account the most recent classification of childhood HNs, by age group, sex, and cancer type. Furthermore, we aim to compare the results with other population-based cancer registries in southern Europe.

## 2 Methods

### 2.1 Study population

Data on childhood HN cases were collected and harmonized from the 15 Spanish population-based cancer registries (PBCRs) belonging to the Spanish Network of Cancer Registries (REDECAN) (14) during the period 1983–2018. These PBCRs cover 17 provinces (Alacant, Albacete, Araba, Asturias, Bizkaia, Castelló, Ciudad Real, Cuenca, Girona, Gipuzkoa, Granada, La Rioja, Murcia, Navarra, Salamanca, Tarragona, and València) and three islands (Mallorca, Las Palmas, and Santa Cruz de Tenerife), representing ~35% of the total Spanish child population (**Table 1**). All the data provided by the registries share the same methodology of data collection, obtained through an active search in different data sources. The data meet the quality controls and follow the procedures and coding rules according to the standards of the International Agency for Research on Cancer (IARC) (15).

**Table 1:** Spanish provinces and islands covered by the cancer registries included in the childhood (0–14 years) hematological neoplasms analysis, period of participation, person-years, number of cases contributing to the incidence analysis, and data quality indicators.

				Quality indicator		
Province/island	Period	Person-years	Number of cases	MV %	NOS %	DCO %
Albacete*	1991–2012	1,378,731	93	97.9	1.1	1.1
Asturias	1991–2013	2,832,009	185	99.5	0.5	0.0
Las Palmas	1993–2015	2,944,080	210	99.5	0.5	0.0
Santa Cruz de Tenerife	1993–2015	2,779,698	168	98.8	0.0	1.2
Ciudad Real*	2004–2012	700,649	35	100.0	0.0	0.0
Cuenca*	1993–2010	528,351	36	97.2	2.9	0.0
Araba	1987–2015	1,243,597	88	97.7	1.1	1.1
Gipuzkoa	1986–2016	3,121,467	235	97.9	1.3	0.9
Bizkaia	1986–2015	4,794,719	342	98.5	0.0	1.5
Girona	1983–2018	3,665,590	246	97.2	1.6	1.2
Granada	1985–2016	4,997,941	281	99.3	0.7	0.0
La Rioja*	1993–2014	906,089	45	91.1	4.4	4.4
Mallorca	1988–2013	2,976,006	181	98.9	1.1	0.0
Murcia	1983–2015	7,872,968	539	97.8	1.5	0.7
Navarra	1983–2015	3,002,901	192	97.9	0.5	1.6
Tarragona	1983–2015	3,567,874	230	99.1	0.0	0.9
Salamanca*	2011–2016	247,071	22	100.0	0.0	0.0
València	1983–2018	13,936,208	915	99.9	0.0	0.1
Castelló	1983–2018	3,016,416	188	100.0	0.0	0.0
Alacant	1983–2018	9,534,776	516	99.8	0.0	0.2
All		74,047,141	4,747	98.4	0.9	0.7

MV, microscopically verified cases; NOS, not otherwise specified cases; DCO, death certificate only cases.

*Provinces/islands excluded in the incidence trend analysis.

A case was defined as any child (0–14 years) diagnosed with an HN who resided in the areas covered by the cancer registries. Standard variables available for each tumor case included basic demographic data (age, sex, and province/island of residence) and tumor data (date of diagnosis, method of diagnosis, tumor histology, and tumor topography). All tumor cases were coded according to the *International Classification of Diseases for Oncology* (ICD-O), third edition, first revision (16). All cases were assembled into four age groups: <1 year, 1–4 years, 5–9 years, and 10–14 years. Diagnoses were grouped according to the most recent child-specific ICCC-3, which divides hematological cancers into two main groups: leukemias and lymphomas. Leukemias are subdivided into lymphoid leukemias (LLs), acute myeloid leukemias (AMLs), chronic myeloproliferative diseases (CMDs), myelodysplastic syndromes and other myeloproliferative diseases (MSs), and unspecified and other specified leukemias. Similarly, lymphomas are grouped into Hodgkin’s lymphoma (HL), non-Hodgkin’s lymphoma (except Burkitt’s lymphoma) (NHL), Burkitt’s lymphoma (BL), miscellaneous lymphoreticular neoplasms (MLNs), and unspecified or other specified lymphomas (1).

### 2.2 Ethics statement

This study was conducted using anonymized data from the participating PBCRs that make up REDECAN. For their part, the cancer registries comply with European and Spanish legislation on the protection of personal data. No intervention was performed on human or animal subjects. Informed consent of the patients is not required for this type of study.

### 2.3 Statistical analysis

Absolute and relative frequencies of all hematological cancers and subgroups were analyzed by age group and sex. HN incidence rates, expressed as crude rates (CRs), age-specific rates, and ASR were estimated by age and sex, expressed per million child-years. The population at risk used was obtained from the National Statistics Institute (INE) (17). ASRs were calculated by the direct method from the summation of the age-specific rates for each age group using the weights of the European population of 2013 (ASR_E_) (18), the ASR_SEGI_ (19), and the new world standard population of the World Health Organization (WHO) (2000–2025) (ASR_WHO_) (20). Incidence sex ratios (ISRs) were calculated as the ratio of the ASR in boys to ASR in girls. In addition, to compare the ASR_SEGI_ from our study with other southern European PBCRs, the ASR_SEGI_ values were obtained from the ICCC, Volume III, published by IARC (1). The countries and time periods included in the comparison were the following: Portugal (1991–2012), Greece (1996–2009), Cyprus (1998–2012), Croatia (2001–2014), France (1993–2012), Bulgaria (1990–2013), Italy (1992–2013), and Malta (1994–2013).

Data normality and homoscedasticity assumptions were checked in order to analyze the incidence trends; to meet these assumptions, trend analyses were restricted to the period 1985–2016. In addition, provinces/islands with shorter time periods, especially those in the first quartile in terms of number of years (<23 years), were excluded from the trend analysis to ensure that most of the provinces/islands contributed to each year of the time trend. Incidence trends were modeled using a simple logarithmic regression model with the ASR_E_ as the dependent variable and time (years) as the independent variable. Changes in the trend were estimated using segmented models, and the APC was calculated for each of the segment trends (21, 22). All the statistical analyses were performed using R version 4.1.3 (23).

## 3 Results

### 3.1 Description of cases

A total of 4,747 childhood HN cases were included in the study of a total population at risk of 74 million child-years. The proportion of the overall microscopically verified cases was 98.4%, with 0.7% of cases based on death certificates only (DCO) (**Table 1**).

**Figure 1:**
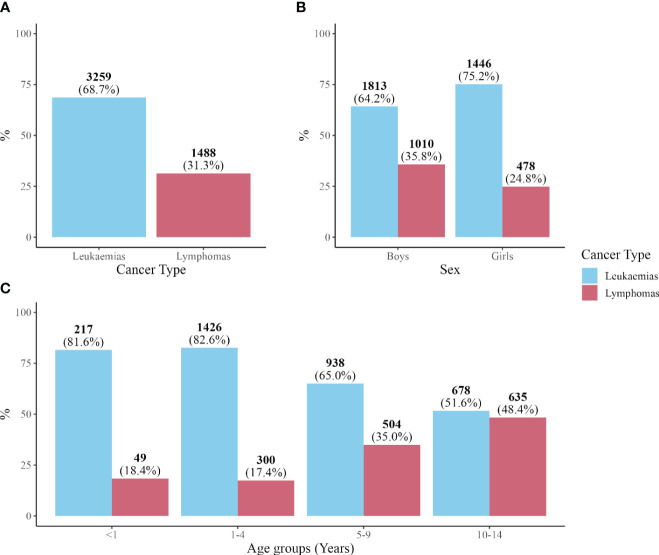
Number of cases and percentage. **(A)** Leukemias and lymphomas. **(B)** Leukemias and lymphomas by sex. **(C)** Leukemias and lymphomas by age group.

**Figure 1** shows the distribution of HNs. Leukemias accounted for two-thirds of HNs diagnosed, while lymphomas accounted for the remaining third. The proportion of leukemias was higher in girls and younger age groups, whereas the proportion of lymphomas was higher in boys and older age groups. A total of 59.5% of patients were boys (N = 2,823), and the age distribution of the groups [n (%)] was as follows: <1 year, 266 (5.6%); 1–4 years, 1,726 (36.4%); 5–9 years, 1,442 (30.4%); and 10–14 years, 1,313 (27.6%).

### 3.2 Incidence

**Supplementary Table S1** shows HN incidence rates for all provinces/islands as a whole and separately for each province/island. Overall, CR and ASR_E_ were 64.1 (95%CI: 62.3; 65.9) and 64.2 (95%CI: 62.4; 66.0) cases of HNs per million child-years, respectively. Higher ASR_E_ values were found in boys than in girls (74.2 (95%CI: 71.5; 77.0) and 53.5 (95%CI: 51.1; 55.9), respectively) with an ISR of 1.4. Differences were found by age group, with the highest age-specific rate of 92.8 cases per million child-years in the 1–4 year age group. The lowest age-specific rate of 49.8 cases per million child-years was observed in the oldest age group (10–14 years).

#### 3.2.1 Leukemias

**Table 2** shows the CR and ASR_E_ for leukemia with 44.0 (95%CI: 42.5; 45.5) and 44.1 (95%CI: 42.6; 45.7) cases per million child-years, respectively. Sex differences in leukemia were smaller compared to those in all HNs, with an ISR of 1.2. Boys had an ASR_E_ of 47.8 (95%CI: 45.6; 50.0), while girls had an ASR_E_ of 40.3 (95%CI: 38.2; 42.4). Age-specific rates revealed a peak incidence of 76.6 (81.9 in boys and 71.0 in girls) cases per million child-years in the age group 1–4 years, while the lowest rate of 25.7 (27.7 in boys and 23.6 in girls) was observed in the age group of 10–14 years.

**Table 2:** Age-specific rates, crude rates, European age-standardized rates, and incidence sex ratios of all the leukemias and leukemia subgroups by sex and age group.

			Age-specific rate									
Sex		N	<1		1–4	5–9	10–14	CR	(CR 95%CI)	ASR_E_	(ASR_E_ 95%CI)	ISR
I. Leukemias, myeloproliferative diseases, and myelodysplastic diseases												
Both		3,259	48.3		76.6	38.2	25.7	44.0	(42.5; 45.5)	44.1	(42.6; 45.7)	1.2
Boys		1,813	44.0		81.9	43.6	27.7	47.6	(45.4; 49.8)	47.8	(45.6; 50.0)	
Girls		1,446	52.9		71.0	32.4	23.6	40.2	(38.1; 42.3)	40.3	(38.2;42.4)	
Ia. Lymphoid leukemias												
Both		2,445	22.3		63.5	30.2	16.0	33.0	(31.7; 34.3)	33.1	(31.8; 34.5)	1.2
Boys		1,368	19.0		68.1	34.6	17.3	35.9	(34.0; 37.8)	36.1	(34.2; 38.0)	
Girls		1,077	25.8		58.6	25.6	14.6	29.9	(28.2; 31.7)	30.1	(28.3; 31.9)	
Ib. Acute myeloid leukemias												
Both		561	16.0		9.5	5.5	6.7	7.6	(7.0; 8.2)	7.6	(7.0; 8.2)	1.1
Boys		306	14.2		10.8	6.3	6.6	8.0	(7.1; 8.9)	8.0	(7.1; 8.9)	
Girls		255	17.9		8.2	4.7	6.7	7.1	(6.2; 8.0)	7.1	(6.2; 8.0)	
Ic. Chronic myeloproliferative diseases												
Both		82	2.2		0.9	0.9	1.2	1.1	(0.9; 1.4)	1.1	(0.9; 1.4)	1.4
Boys		49	1.7		0.9	1.0	1.7	1.3	(0.9; 1.7)	1.3	(0.9; 1.6)	
Girls		33	2.8		0.9	0.8	0.7	0.9	(0.6; 1.2)	0.9	(0.6; 1.2)	
Id. Myelodysplastic syndromes												
Both		63	3.8		1.2	0.5	0.5	0.9	(0.6; 1.1)	0.9	(0.6; 1.1)	1.1
Boys		34	5.2		0.8	0.6	0.5	0.9	(0.6; 1.2)	0.9	(0.6; 1.2)	
Girls		29	2.3		1.7	0.3	0.4	0.8	(0.5; 1.1)	0.8	(0.5; 1.1)	
Ie. Unspecified and other specified leukemias												
Both		108	4.0		1.6	1.1	1.4	1.5	(1.2; 1.7)	1.5	(1.2; 1.7)	1.0
Boys		56	3.9		1.3	1.1	1.6	1.5	(1.1; 1.9)	1.5	(1.1; 1.9)	
Girls		52	4.1		1.7	1.0	1.3	1.5	(1.1; 1.8)	1.5	(1.1; 1.8)	

CR, crude rate; ASR_E_, age-standardized rate using the 2013 European population; CI, confidence interval; ISR, incidence sex ratio.

Differences between leukemia subgroups were observed, with LL having the highest ASR_E_ of 33.1 (95%CI: 31.8; 34.5) cases per million child-years, representing 75.0% of all leukemia cases. AML was the second most common subtype with an ASR_E_ of 7.6 (95%CI: 7.0; 8.2) cases per million child-years, accounting for 17.2% of all leukemia cases. The remaining leukemia subtypes accounted for less than 8.0% of all leukemia cases, as shown in **Supplementary Figure S1** and **Table 2**.

Sex differences were also observed between leukemia subtypes, with LL reporting higher differences with an ISR of 1.2, compared to AML with an ISR of 1.1. Furthermore, age group differences within the leukemia subtypes revealed a peak at the age of 1–4 years with an age-specific rate of 63.5 corresponding to LL. In contrast, the remaining subtypes showed a peak in the age group of <1 year with age-specific rates of 16.0, 2.2, 3.8, and 4.0 per million child-years, corresponding to AML, CMDs, MSs, and unspecified and other specified leukemias, respectively.

**Table 3** shows the differences in leukemia incidence rates between southern European countries, using the latest ICCC-3 from 2017. The results revealed that Western countries (Portugal, Spain, and France) had an ASR_SEGI_ below 50 cases per million child-years. Conversely, Eastern countries (Greece, Malta, Italy, Cyprus, and Croatia) exhibited an ASR_SEGI_ above 50 cases, with the exception of Bulgaria, which reported 43.4 cases. For further reference, the ASR_WHO_ values for Spain are depicted in **Supplementary Table S2**.

**Table 3:** Comparison of world Segi 1960 age-standardized rates between southern European countries (1).

ASR_SEGI_														
Spain		Portugal		Greece		Cyprus (south-west)			Croatia		France	Bulgaria	Italy	Malta
I. Leukemias, myeloproliferative diseases, and myelodysplastic diseases														
47.3		44.6		51.9		58.0			60.3		45.2	43.4	58.4	60.7
Ia. Lymphoid leukemias														
35.8		32.3		45.2		44.5			50.5		34.5	32.5	47.1	46.1
Ib. Acute myeloid leukemias														
7.9		8.2		5.5		10.1			7.7		6.9	4.8	7.7	7.5
Ic. Chronic myeloproliferative diseases														
1.1		1.3		0.5		0.4			0.8		1.0	1.0	1.6	3.8
Id. Myelodysplastic syndrome														
1.0		1.5		0.1		1.3			0.3		1.6	0.4	0.7	2.7
Ie. Unspecified and other specified leukemias														
1.5		1.2		0.5		1.6			1.0		1.2	4.7	1.2	0.7
II. Lymphomas and reticuloendothelial neoplasms														
19.4		22.8		15.9		23.8			23.1		20.0	18.1	25.7	21.8
IIa. Hodgkin lymphomas														
5.3		8.7		6.1		12.1			7.2		7.1	8.2	10.3	6.8
IIb. Non-Hodgkin lymphomas (except Burkitt’s lymphoma)														
5.8		6.7		5.3		9.2			7.8		6.2	6.0	7.4	6.0
IIc. Burkitt’s lymphoma														
5.4		4.6		4.5		1.8			2.9		4.9	1.7	4.6	3.9
IId. Miscellaneous lymphoreticular neoplasms														
2.3		2.1		0.0		0.0			5.1		1.5	1.4	2.6	5.1
IIe. Unspecified and other specified lymphomas														
0.4		0.7		0.0		0.8			0.2		0.2	0.8	0.8	0.0

ASR_SEGI_, age-standardized rate using the 1960 Segi world population.

#### 3.2.2 Lymphomas

**Table 4** shows the CR and ASR_E_ of lymphomas with 20.1 (95%CI: 19.1; 21.1) and 20.0 (95%CI: 19.0; 21.1) cases per million child-years, respectively. Higher sex differences were observed in lymphomas with an ISR of 2.0. Boys had an ASR_E_ of 26.5 (95%CI: 24.9; 28.1), while girls had an ASR_E_ of 13.2 (95%CI: 12.0; 14.4) cases per million child-years. Age-specific rates revealed an increase in lymphoma incidence with age, with the highest rate of 24.1 (30.4 in boys and 17.4 in girls) cases per million child-years in the age group 10–14 years and the lowest rate of 10.9 (11.7 in boys and 10.1 in girls) in the age group <1 year.

**Table 4:** Age-specific rates, crude rates, European age-standardized rates, and incidence sex ratio of all lymphomas and lymphoma subgroups by sex and age group.

		Age-specific rate								
Sex	N	<1	1–4	5–9	10–14	CR	(CR 95%CI)	ASR_E_	(ASR_E_ 95%CI)	ISR
II. Lymphomas and reticuloendothelial neoplasms										
Both	1,488	10.9	16.1	20.5	24.1	20.1	(19.1; 21.1)	20.0	(19.0; 21.1)	2.0
Boys	1,010	11.7	22.2	28.4	30.4	26.5	(24.9; 28.2)	26.5	(24.9; 28.1)	
Girls	478	10.1	9.6	12.2	17.4	13.3	(12.1; 14.5)	13.2	(12.0; 14.4)	
IIa. Hodgkin lymphomas										
Both	449	0.0	1.0	5.2	11.5	6.1	(5.5; 6.6)	6.0	(5.4; 6.5)	1.5
Boys	276	0.0	1.8	7.2	12.4	7.3	(6.4; 8.1)	7.2	(6.3; 8.0)	
Girls	173	0.0	0.2	3.1	10.4	4.8	(4.1; 5.5)	4.7	(4.0; 5.4)	
IIb. Non-Hodgkin lymphomas (except Burkitt’s lymphoma)										
Both	443	1.6	4.9	6.8	6.8	6.0	(5.4; 6.5)	6.0	(5.4; 6.5)	2.3
Boys	312	1.7	6.5	9.5	9.3	8.2	(7.3; 9.1)	8.2	(7.3; 9.1)	
Girls	131	1.4	3.2	3.9	4.1	3.6	(3.0; 4.3)	3.6	(3.0; 4.3)	
IIc. Burkitt’s lymphoma										
Both	393	0.2	6.6	6.3	4.4	5.3	(4.8; 5.8)	5.3	(4.8; 5.9)	3.6
Boys	312	0.4	10.8	9.3	6.7	8.2	(7.3; 9.1)	8.2	(7.3; 9.1)	
Girls	81	0.0	2.1	3.1	2.0	2.3	(1.8; 2.7)	2.3	(1.8; 2.8)	
IId. Miscellaneous lymphoreticular neoplasms										
Both	172	8.9	3.4	1.7	1.0	2.3	(2.0; 2.7)	2.3	(2.0; 2.7)	1.1
Boys	92	9.5	3.1	1.7	1.4	2.4	(1.9; 2.9)	2.4	(1.9; 2.9)	
Girls	80	8.3	3.8	1.7	0.6	2.2	(1.7; 2.7)	2.3	(1.8; 2.7)	
IIe. Unspecified and other specified lymphomas										
Both	31	0.2	0.2	0.6	0.4	0.4	(0.3; 0.6)	0.4	(0.3; 0.6)	1.3
Boys	18	0.0	0.1	0.7	0.6	0.5	(0.3; 0.7)	0.5	(0.3; 0.7)	
Girls	13	0.5	0.3	0.5	0.2	0.4	(0.2; 0.6)	0.4	(0.2; 0.6)	

CR, crude rate; ASR_E_, age-standardized rate using the 2013 European population; CI, confidence interval; ISR, incidence sex ratio.

Similar incidence rates were estimated for HL, NHL, and BL, reporting ASR_E_ values of 6.0 (95%CI: 5.4; 6.5), 6.0 (95%CI: 5.4; 6.5), and 5.3 (95%CI: 4.8; 5.9), respectively, representing 86.0% of all lymphoma cases. The remaining 14.0% of cases were predominantly MLN, as shown in **Supplementary Figure S2** and **Table 4**.

Differences in incidence by sex and age group were observed between lymphoma subtypes. The incidence of MLN decreased with age, and girls showed similar incidence rates to boys with an ISR of 1.1. In contrast, BL had the highest sex difference of all HNs, with an ISR of 3.6. In addition, HL incidence rates increased dramatically with age, with no cases in the age group of <1 year and 11.5 cases in the age group of 10–14 years.

**Table 3** shows lymphoma ASR_SEGI_ among southern European countries. The majority of these countries exhibited incidence rates of approximately 20 cases per million child-years. In particular, Italy and Cyprus demonstrated the highest rates with 25.7 and 23.8 cases, respectively. In contrast, Greece displayed the lowest at 15.9 cases per million child-years. In addition, **Supplementary Table S3** provides the ASR_WHO_ for Spain for further reference.

### 3.3 Incidence trends

**Figure 2:**
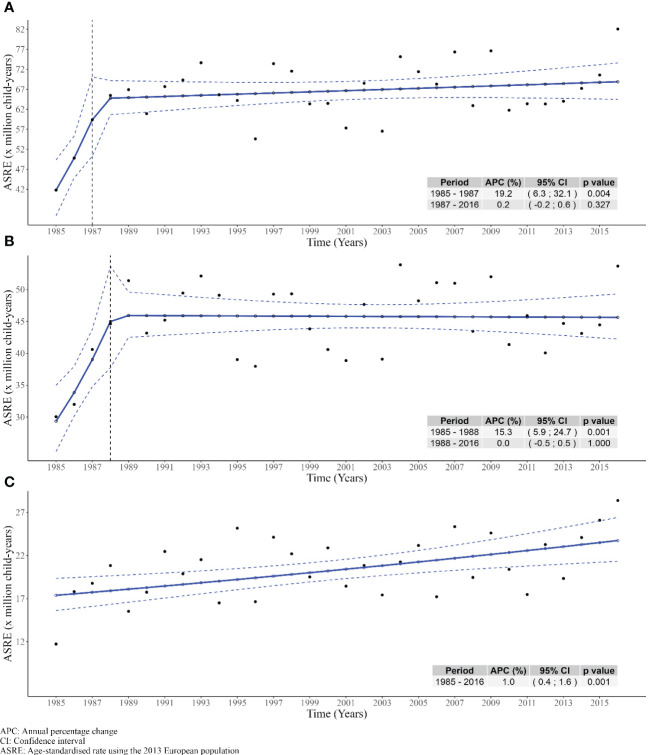
Trends of age-standardized rates using the 2013 European standard population during the period 1985-2016 in Spain. (A) All hematological neoplasms. (B) Leukemias. (C) Lymphomas.

ASR_E_ by diagnosis period (1983–1994, 1995–2006, and 2007–2018) was calculated as shown in **Supplementary Table S4** and revealed statistically significant differences between the first and last periods for all HNs and lymphomas. **Figure 2** depicts the differences in trend patterns between childhood leukemias and lymphomas. Leukemias showed a statistically significant increase in the first 3 years (1985–1988) with an APC of 15.3% (95%CI: 5.9; 24.7), followed by a stable period between 1988 and 2016 (APC: 0.0% (95%CI: −0.5; 0.7). In contrast, childhood lymphomas showed no change in trend over the period, with a statistically significant steady increase in the APC of 1.0% (95%CI: 0.4; 1.6). Additional incidence trend analyses were performed by sex and age group for HNs, leukemia, and lymphoma, as shown in **Supplementary Table S5**.

## 4 Discussion

Information on the epidemiology of childhood HNs is scarce, as most studies focus on adults due to their higher incidence compared to children (6). Therefore, this study, using data from 15 PBCRs, allows us to provide a broad descriptive analysis of the incidence and incidence trends of all childhood HNs over 36 years, from 1983 to 2018 in Spain. In addition, the long time period of this study and the increased population coverage allow us to update the epidemiological results of these malignancies since previous publications in Spain covered a smaller population area and a shorter time period until 2007 (12, 13).

New histology codes created after the 2005 ICCC-3, due to multiple new diagnostic methods in progress using molecular techniques, were accurately grouped according to the updated version, the 2017 ICCC-3 (5). The latest classification is not widely used in other studies due to its recent publication. At the national level in Spain, previous publications on HNs in children have used the ICCC-3 of 2005 (12, 13, 24). Therefore, the analysis of incidence and incidence trends not only provides insight over a long period of time but also provides more recent results in terms of histological aggregation. At the international level, only one study was found that used the ICCC-3 of 2017 (25), since the most recent studies used the ICCC-3 of 2005 (26–28). Therefore, we suggest the use of this more recent classification for future studies in European PBCRs. Furthermore, ASR exhibited variability between the different standard populations used. In particular, the use of the Segi 1960 standard population yielded the highest incidence rates due to its emphasis on younger age groups (20). Conversely, the ASR_E_ and the ASR_WHO_ demonstrated comparable rates, suggesting that the new WHO standard reflected the study population structure more accurately. In light of these findings, we recommend adopting the new WHO standard to be used for international comparisons.

Overall, we reported that two-thirds of all childhood HNs were leukemias and the remaining third were lymphomas. Similar proportions have been reported in southern and eastern Europe (29). An ASR_E_ of 64 cases per million child-years of HNs was observed during the study period, with a statistically significant increase in incidence during the first 3 years, followed by stable rates for the remaining years. To the best of our knowledge, no publication in the literature provides an overview of the incidence and incidence trends of HNs as a whole. Most of the authors report the incidence and incidence trends of HNs divided into two large groups: leukemias and lymphomas (6, 7, 30). Differences between sexes and age groups have been observed, with boys and children aged 1–4 years tending to have higher incidence rates. Furthermore, the sex differences in HNs are greater than in other cancers, but the endogenous causes are largely unknown despite all the research that has been performed (31, 32).

Lymphoid leukemias were the main subgroup of all leukemias, followed by a smaller proportion of AML. An ASR_SEGI_ of 47.3 cases of childhood leukemia per million child-years was observed, with similar results to those previously reported in Spain and other European regions (12, 24, 30). These results contrast with the lower values reported in developing countries, such as sub-Saharan Africa and South Asia (6). The peak in leukemia incidence in the age group 1–4 years, specifically observed in LL, is a pattern that has been reported in previous studies (12, 24, 33). At present, this peak was first identified in the early 20th century, and it was suggested that the risk factors triggering this increase were unknown (34). However, subsequent studies have suggested that benzene, agricultural exposures, smoking and alcohol consumption, cigarettes, and illicit drugs during pregnancy are predisposing factors for childhood LL (35). Meanwhile, the reported incidence peak at the age of <1 year, corresponding to AML, is mainly attributed to hereditary conditions such as Down syndrome and Fanconi anemia (36). From an international perspective, the leukemia incidence patterns observed in this study by age group, sex, and subtype were similar to those in France and Portugal (1, 30, 33). In addition, Surveillance, Epidemiology, and End Results (SEER) leukemia incidence rates were similar to those observed in this study, whereas Cancer Research UK incidence rates were higher (37, 38).

Similar proportions were observed for HL, NHL, and BL, with ASR_E_ values of 6.0, 5.4, and 6.0 cases per million child-years, respectively, out of a total of 20 cases per million child-years for all lymphomas. The most recent study published in Spain reported lower incidence values for lymphomas as a whole, but this can be attributed to the exclusion of MLN and unspecified and other specified lymphomas subtypes from the incidence analysis (13). Internationally, southern Europe, and therefore the region included in this study, has one of the highest lymphoma incidence rates in the world, with the exception of other Mediterranean regions such as North African countries (6, 7). It has been suggested that the elevated incidence rates observed in the Mediterranean region may be due to environmental factors, as different ethnic groups living in the region, including Caucasians, Arabs, and Jews, exhibit higher incidence rates of these malignancies (39). Aside from this region, Cancer Research UK lymphoma incidence rates were similar to those observed in this study, while SEER results were higher, specifically for HL (37, 38).

Sex and age differences were also observed in our results, which have been widely reported by other authors (6, 12, 13). Although the main risk factors for these sex differences are currently unknown, some authors suggest that boys have an innate susceptibility associated with immune surveillance that makes them more vulnerable to proto-oncogenic mutations (6, 31, 40). The association between lymphoma incidence and age is due to the fact that these malignancies are more common in adolescents, with the age group of 10–14 years having the highest age-specific incidence rate of all age groups (13, 41).

Our findings showed that the overall incidence trends of HNs were similar to those previously reported for leukemias in Spain, with a statistically significant increase in the early years of the period, followed by a stabilization since 1989 (12). We suggest that this steep increase in leukemia incidence during 1985–1988 is probably an artifact due to the lack of cases registered by the provinces/islands during the first years of the study. In our study, the incidence rates were found to be stable in most recent years added. Meanwhile, other studies have shown different patterns of leukemia incidence trends, with some reporting a steady statistically significant increase without a stabilization (8, 10, 26, 27). However, others reported a stabilization over the whole period of the study (9, 42, 43), especially for LL and AML in Germany for the most recent period available in the study (28).

This heterogeneity in incidence trends was also observed for lymphomas, as our results revealed a statistically significant steady increase of 1% per year, in contrast to previous findings in southern Europe and Spain, where no increase was detected (8, 13). However, a similar increase was observed in Denmark and Australia for the periods 1977–2014 and 1983–2015, respectively (9, 26). We hypothesized that the increase in lymphoma incidence could be due to an increase in environmental risk factors in the Mediterranean region, as these have been suggested to be the main risk factors for these malignancies in this area (39). However, other countries, such as Switzerland, Italy, Japan, and the England, reported a stabilization since the beginning of the period analyzed (10, 42, 43). Many factors may contribute to the differences in incidence trend results, despite all the standardized definitions of quality criteria by IARC. One factor that may contribute to these differences is the use of different versions of the ICCC in the histological grouping, as this may complicate the interpretation of incidence trends (44).

Despite all the studies discussed on incidence trends, the number of collaborative studies is scarce. One of the reasons contributing to this may be the implementation of the General Data Protection Regulation in Europe since 2018, which, among other things, may affect researchers and make data sharing more difficult than before (45).

## 5 Strengths and limitations

Our results are strengthened by the use of population-based incidence data from REDECAN, in which more than 95% of cases were verified microscopically. In addition, the large area covered in Spain and the long time period allowed us to perform the main analysis with high statistical power. Furthermore, few studies grouped the histology codes according to the latest ICCC-3 of 2017 and provided the ASR using the new WHO world standard population (2000–2025). However, the sample size was limited when performing all sub-analyses involving less common pathologies, such as calculating the incidence of specific sex, age group, and HN subgroup. Another limitation of the study was the lack of period homogeneity between cancer registries, but this was addressed by ensuring that most of the provinces/islands contributed to each year of the trend. In addition, this study does not represent the entire Spanish child population, as incidence rates may differ between the covered and non-covered areas, as a previous study has revealed differences in incidence rates between urban and rural areas in Spain (46). Finally, changes in histological grouping and diagnostic criteria throughout the period could have also affected the results obtained and the comparisons with previous studies.

## 6 Conclusions

This study presents an updated population-based analysis of the incidence and incidence trends of childhood HNs in Spain covering a long period from 1983 to 2018 and a large area. Our results showed that leukemias are the most common HNs in children, and their incidence has remained stable since 1988, while lymphomas are less common but their incidence is increasing every year. The observed incidence rates of lymphomas are similar to those reported in other southern European countries, while the similarities in leukemia incidence rates were limited to the southwestern European countries. Furthermore, we recommend the use of the new ICCC-3 from 2017 and the use of the new WHO world standard population (2000–2025) in future studies conducted by European PBCRs. Collaborative cancer registry projects, such as the REDECAN, provide the opportunity to assess epidemiological indicators of less common cancers, such as pediatric HNs. Consequently, all these reported epidemiological findings could help health authorities and clinicians to have updated results of these malignancies for the more recent years in Spain. Furthermore, the results suggest the need for more studies focusing on the risk factors of childhood lymphomas, as their incidence is increasing every year in Spain.

## REDECAN working group

*REDECAN Working Group*: **Girona Cancer Registry** – Coordination of hematology neoplasms in REDECAN: Rafael Marcos Gragera, Montse Puigdemont, Anna Vidal Vila, Arantza Sanvisens, and Jan Trallero; **Tarragona Cancer Registry** – Data coordination in REDECAN: Marià Carulla, Alberto Ameijide, Clàudia Pla, and Jaume Galceran; **Álava Cancer Registry**: Arantza López de Munain, Patricia Sancho, and María Luisa Iruretagoyena; **Albacete Cancer Registry**: Cristina Ramírez; **Asturias Cancer Registry**: Susana Merino Perera, Virginia Menéndez García, and Marta Rodríguez Camblor; **Bizkaia Cancer Registry**: Visitación de Castro, Marta De La Cruz, and Joseba Bidaurrazaga; **Castellón Cancer Registry**: Consol Sabater Gregori, Isabel Sáez Lloret, Ana Vizcaíno Batllés, and Xavier Peñalver Herrero; **Ciudad Real Cancer Registry**: Cristina Díaz del Campo; **Cuenca Cancer Registry**: Ana Isabel Marcos and Rosario Jimenez Chillarón; **Gipuzkoa Cancer Registry**: Amaia Aizpurua; **Canary Islands Cancer Registry**: María Dolores Rojas Martín, María Araceli Alemán Herrera, Emilio De Miguel, María Magdalena Ramos Marrero, María Carmen Gábas, Beatriz Hidalgo Martín, and Olga Leonor Velázquez Castilla; **Granada Cancer Registry**: María José Sánchez, Daysi Yoe-Ling Chang-Chan, and Miguel Rodríguez Barranco; **La Rioja Cancer Registry**: María Isabel Palacios and Enrique Ramalle; **Mallorca Cancer Registry**: Paula Franch, Patricia Ruiz Armengol, and Carmen Sánchez Contador; **Murcia Cancer Registry**: María Dolores Chirlaque, Antonia Sánchez Gil, and Ricardo José Vaamonde; **Navarra Cancer Registry**: Marcela Guevara and Eva Ardanaz; **Castilla y León Cancer Registry**: Pilar Gutiérrez, Rufino Álamo, and Enrique Cabrera; **Registro Español de Tumores Infantiles**: Adela Cañete, Elena Pardo, and Rafael Peris Bonet; **Registro de Tumores Infantiles y Adolescentes de la Comunitat Valenciana**: Ana Vizcaino, Fernando Almela Vich, and Noura Jeghalef El Karoni.

## Data availability statement

The raw data supporting the conclusions of this article will be made available by the authors, without undue reservation.

## Ethics statement

The studies involving human participants were reviewed and approved by CEIM Girona Institut d’Investigació Biomèdica de Girona Dr. Josep Trueta (IDIBGI). Written informed consent from the participants’ legal guardian/next of kin was not required to participate in this study in accordance with the national legislation and institutional requirements.

## Author contributions

JT, AS, and RM-G contributed to the study conception and design. Data collection was performed by FA, NJ, IS, CD-D-C, AM-N, AAA, PS, MO, MS, JP, PF, MC, MG, CR, MC, MA, PG, and RM-G. Data analysis was performed by JT, AS, and AA. The first draft of the manuscript was written by JT. All authors contributed to the article and approved the submitted version.
