# Supplementary material for: Incidence and time trends of childhood hematological neoplasms: a 36-year population-based study in the southern European context, 1983–2018
Source: Front Oncol. 2023 Jul 25;13:1197850. doi: 10.3389/fonc.2023.1197850 (PMC10408119; doi:10.3389/fonc.2023.1197850)
Supplement: Supplementary file 1 [file DataSheet_1.docx]

# Supplementary material

Table S1: Age-specific rate, crude rates, 2013 European age-standardised rates and incidence sex ratios of all the Spanish provinces/islands by sex and age groups for all haematological neoplasms.

|  | | **Age-specific rate** | | | |  | | | | |
| --- | --- | --- | --- | --- | --- | --- | --- | --- | --- | --- |
| **Sex** | **N** | **<1** | **1-4** | **5-9** | **10-14** | **CR** | **(CR 95% CI)** | **ASR** | **(ASR 95% CI)** | **ISR** |
| **Overall** | | | | | | | | | | |
| Both | 4,747 | 59.23 | 92.75 | 58.71 | 49.76 | 64.11 | (62.28;65.93) | 64.18 | (62.36;66) | 1.39 |
| Boys | 2,823 | 55.67 | 104.13 | 71.99 | 58.11 | 74.15 | (71.41;76.89) | 74.23 | (71.49;76.97) |  |
| Girls | 1,924 | 63.03 | 80.66 | 44.65 | 40.96 | 53.48 | (51.09;55.87) | 53.53 | (51.14;55.92) |  |
| **Albacete** | | | | | | | | | | |
| Both | 93 | 35.48 | 109.71 | 64.11 | 46.42 | 67.45 | (53.75;81.16) | 67.64 | (53.9;81.38) | 2.1 |
| Boys | 64 | 22.89 | 151.31 | 94.61 | 54.87 | 90.16 | (68.07;112.25) | 90.64 | (68.43;112.85) |  |
| Girls | 29 | 48.93 | 65.50 | 31.85 | 37.45 | 43.35 | (27.58;59.13) | 43.26 | (27.52;59) |  |
| **Asturias** | | | | | | | | | | |
| Both | 185 | 30.29 | 78.63 | 62.77 | 64.52 | 65.32 | (55.91;74.74) | 65.31 | (55.9;74.72) | 1.65 |
| Boys | 117 | 47.10 | 78.11 | 94.34 | 75.59 | 80.54 | (65.95;95.14) | 80.88 | (66.22;95.54) |  |
| Girls | 68 | 12.48 | 79.19 | 29.43 | 52.91 | 49.30 | (37.58;61.01) | 48.88 | (37.26;60.5) |  |
| **Las Palmas** | | | | | | | | | | |
| Both | 210 | 45.47 | 94.68 | 61.11 | 68.77 | 71.33 | (61.68;80.98) | 71.16 | (61.54;80.78) | 1.38 |
| Boys | 124 | 55.34 | 100.35 | 73.91 | 82.58 | 82.53 | (68;97.06) | 82.34 | (67.86;96.82) |  |
| Girls | 86 | 35.05 | 88.72 | 47.79 | 54.46 | 59.66 | (47.04;72.27) | 59.52 | (46.94;72.1) |  |
| **Santa Cruz de Tenerife** | | | | | | | | | | |
| Both | 168 | 11.78 | 77.70 | 56.78 | 59.82 | 60.44 | (51.3;69.58) | 60.24 | (51.13;69.35) | 1.55 |
| Boys | 104 | 22.80 | 104.22 | 70.20 | 61.46 | 72.89 | (58.88;86.9) | 72.74 | (58.77;86.71) |  |
| Girls | 64 | 0.00 | 49.53 | 42.63 | 58.09 | 47.30 | (35.72;58.89) | 47.01 | (35.49;58.53) |  |
| **Ciudad Real** | | | | | | | | | | |
| Both | 35 | 87.77 | 38.22 | 53.11 | 48.79 | 49.95 | (33.4;66.51) | 50.07 | (33.49;66.65) | 0.91 |
| Boys | 17 | 42.63 | 21.15 | 68.60 | 47.44 | 47.08 | (24.7;69.45) | 47.84 | (25.1;70.58) |  |
| Girls | 18 | 135.66 | 56.44 | 36.58 | 50.21 | 53.01 | (28.51;77.52) | 52.42 | (28.19;76.65) |  |
| **Cuenca** | | | | | | | | | | |
| Both | 36 | 32.12 | 137.68 | 62.53 | 31.48 | 68.14 | (45.88;90.39) | 68.74 | (46.28;91.2) | 0.69 |
| Boys | 15 | 0.00 | 135.59 | 44.77 | 20.60 | 55.83 | (27.58;84.08) | 56.37 | (27.85;84.89) |  |
| Girls | 21 | 65.48 | 139.83 | 80.87 | 42.79 | 80.87 | (46.29;115.45) | 81.56 | (46.67;116.45) |  |
| **Araba** | | | | | | | | | | |
| Both | 88 | 38.58 | 99.70 | 64.32 | 62.13 | 70.76 | (55.97;85.55) | 70.80 | (56;85.6) | 2.29 |
| Boys | 62 | 24.98 | 144.06 | 96.73 | 77.99 | 97.29 | (73.08;121.51) | 97.64 | (73.34;121.94) |  |
| Girls | 26 | 53.01 | 52.88 | 30.39 | 45.48 | 42.88 | (26.4;59.36) | 42.61 | (26.22;59) |  |
| **Gipuzkoa** | | | | | | | | | | |
| Both | 235 | 72.97 | 118.54 | 61.83 | 58.17 | 75.29 | (65.66;84.91) | 75.45 | (65.81;85.09) | 1.73 |
| Boys | 152 | 60.80 | 143.03 | 84.11 | 77.15 | 94.78 | (79.72;109.85) | 94.99 | (79.9;110.08) |  |
| Girls | 83 | 85.87 | 92.70 | 38.32 | 38.10 | 54.68 | (42.92;66.45) | 54.81 | (43.01;66.61) |  |
| **Bizkaia** | | | | | | | | | | |
| Both | 342 | 49.28 | 109.21 | 58.19 | 61.67 | 71.33 | (63.77;78.89) | 71.58 | (63.99;79.17) | 1.39 |
| Boys | 203 | 54.78 | 125.65 | 69.81 | 69.99 | 82.53 | (71.18;93.89) | 82.89 | (71.48;94.3) |  |
| Girls | 139 | 43.46 | 91.87 | 45.95 | 52.91 | 59.52 | (49.63;69.42) | 59.67 | (49.75;69.59) |  |
| **Girona** | | | | | | | | | | |
| Both | 246 | 95.27 | 96.08 | 57.94 | 49.13 | 67.11 | (58.72;75.5) | 66.78 | (58.43;75.13) | 1.5 |
| Boys | 151 | 75.77 | 123.19 | 74.59 | 53.89 | 80.09 | (67.32;92.86) | 79.70 | (66.98;92.42) |  |
| Girls | 95 | 115.93 | 67.37 | 40.31 | 44.08 | 53.37 | (42.64;64.1) | 53.10 | (42.42;63.78) |  |
| **Granada** | | | | | | | | | | |
| Both | 281 | 39.94 | 84.20 | 55.99 | 39.67 | 56.22 | (49.65;62.8) | 56.43 | (49.82;63.04) | 1.25 |
| Boys | 160 | 25.80 | 93.38 | 65.53 | 43.53 | 62.23 | (52.59;71.87) | 62.45 | (52.77;72.13) |  |
| Girls | 121 | 55.02 | 74.44 | 45.89 | 35.60 | 49.86 | (40.97;58.74) | 50.06 | (41.14;58.98) |  |
| **La Rioja** | | | | | | | | | | |
| Both | 45 | 50.04 | 58.56 | 37.10 | 54.74 | 49.66 | (35.15;64.18) | 49.34 | (34.91;63.77) | 1.32 |
| Boys | 26 | 65.17 | 57.18 | 46.28 | 62.87 | 56.10 | (34.55;77.66) | 55.89 | (34.41;77.37) |  |
| Girls | 19 | 34.18 | 60.00 | 27.54 | 46.20 | 42.92 | (23.63;62.21) | 42.48 | (23.37;61.59) |  |
| **Mallorca** | | | | | | | | | | |
| Both | 181 | 51.12 | 81.55 | 51.44 | 55.69 | 60.82 | (51.96;69.68) | 60.41 | (51.61;69.21) | 1.58 |
| Boys | 113 | 39.62 | 93.77 | 65.99 | 72.40 | 73.80 | (60.19;87.41) | 73.49 | (59.95;87.03) |  |
| Girls | 68 | 63.38 | 68.50 | 36.02 | 38.10 | 47.06 | (35.88;58.25) | 46.56 | (35.49;57.63) |  |
| **Murcia** | | | | | | | | | | |
| Both | 539 | 71.34 | 94.83 | 69.13 | 47.82 | 68.46 | (62.68;74.24) | 68.37 | (62.61;74.13) | 1.27 |
| Boys | 309 | 75.01 | 92.86 | 83.76 | 56.76 | 76.18 | (67.68;84.67) | 76.21 | (67.7;84.72) |  |
| Girls | 230 | 67.42 | 96.93 | 53.58 | 38.34 | 60.26 | (52.48;68.05) | 60.04 | (52.28;67.8) |  |
| **Navarra** | | | | | | | | | | |
| Both | 192 | 64.29 | 92.49 | 64.37 | 43.18 | 63.94 | (54.89;72.98) | 64.11 | (55.04;73.18) | 1.43 |
| Boys | 116 | 51.89 | 97.46 | 78.16 | 60.20 | 75.02 | (61.37;88.68) | 75.17 | (61.49;88.85) |  |
| Girls | 76 | 77.52 | 87.21 | 49.75 | 25.14 | 52.17 | (40.44;63.9) | 52.39 | (40.61;64.17) |  |
| **Registry: Tarragona** | | | | | | | | | | |
| Both | 230 | 86.06 | 83.04 | 58.82 | 52.34 | 64.46 | (56.13;72.79) | 64.35 | (56.04;72.66) | 1.38 |
| Boys | 137 | 78.77 | 90.94 | 70.19 | 65.74 | 74.51 | (62.03;86.99) | 74.38 | (61.93;86.83) |  |
| Girls | 93 | 93.88 | 74.61 | 46.76 | 38.15 | 53.78 | (42.85;64.71) | 53.71 | (42.79;64.63) |  |
| **Salamanca** | | | | | | | | | | |
| Both | 22 | 138.69 | 110.47 | 58.66 | 95.18 | 89.04 | (51.85;126.24) | 89.17 | (51.91;126.43) | 3.2 |
| Boys | 17 | 267.77 | 152.14 | 67.83 | 160.39 | 132.60 | (69.58;195.62) | 133.22 | (69.89;196.55) |  |
| Girls | 5 | 0.00 | 65.57 | 48.77 | 24.75 | 42.06 | (5.22;78.91) | 41.66 | (5.15;78.17) |  |
| **València** | | | | | | | | | | |
| Both | 915 | 74.10 | 97.94 | 61.34 | 45.90 | 65.66 | (61.4;69.91) | 65.98 | (61.71;70.25) | 1.29 |
| Boys | 530 | 65.74 | 110.34 | 72.08 | 51.49 | 73.87 | (67.58;80.16) | 74.17 | (67.86;80.48) |  |
| Girls | 385 | 83.06 | 84.73 | 49.95 | 39.98 | 56.94 | (51.25;62.63) | 57.29 | (51.57;63.01) |  |
| **Castelló** | | | | | | | | | | |
| Both | 188 | 16.40 | 87.79 | 57.47 | 56.55 | 62.33 | (53.42;71.23) | 62.17 | (53.29;71.05) | 1.53 |
| Boys | 116 | 21.18 | 107.01 | 69.56 | 66.28 | 74.96 | (61.32;88.6) | 74.77 | (61.17;88.37) |  |
| Girls | 72 | 11.30 | 67.44 | 44.74 | 46.34 | 49.02 | (37.7;60.34) | 48.88 | (37.59;60.17) |  |
| **Alacant** | | | | | | | | | | |
| Both | 516 | 59.00 | 88.40 | 47.48 | 35.60 | 54.12 | (49.45;58.79) | 54.35 | (49.67;59.03) | 1.21 |
| Boys | 290 | 58.80 | 93.88 | 54.65 | 38.96 | 59.11 | (52.3;65.91) | 59.32 | (52.5;66.14) |  |
| Girls | 226 | 59.22 | 82.56 | 39.89 | 32.06 | 48.83 | (42.46;55.2) | 49.07 | (42.68;55.46) |  |
| CR: Crude rate | | | | | | | | | | |
| ASR: European age-standardised rate | | | | | | | | | | |
| CI: Confidence interval  ISR: Incidence sex ratio | | | | | | | | | | |


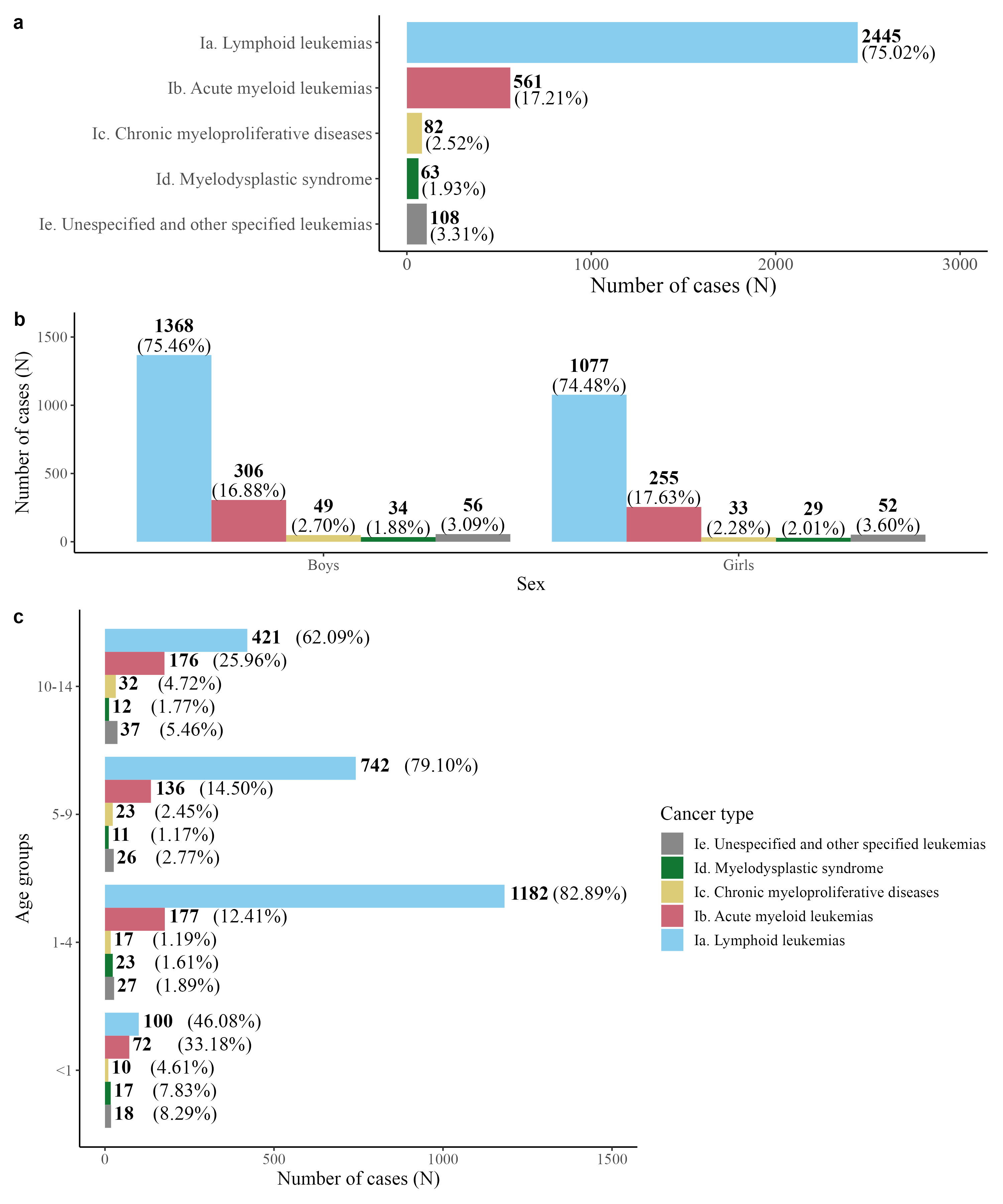


Figure S1: Distribution of a) Leukaemias subgroups, b) Leukaemias subgroups by sex and c) Leukaemias subgroups by age groups.


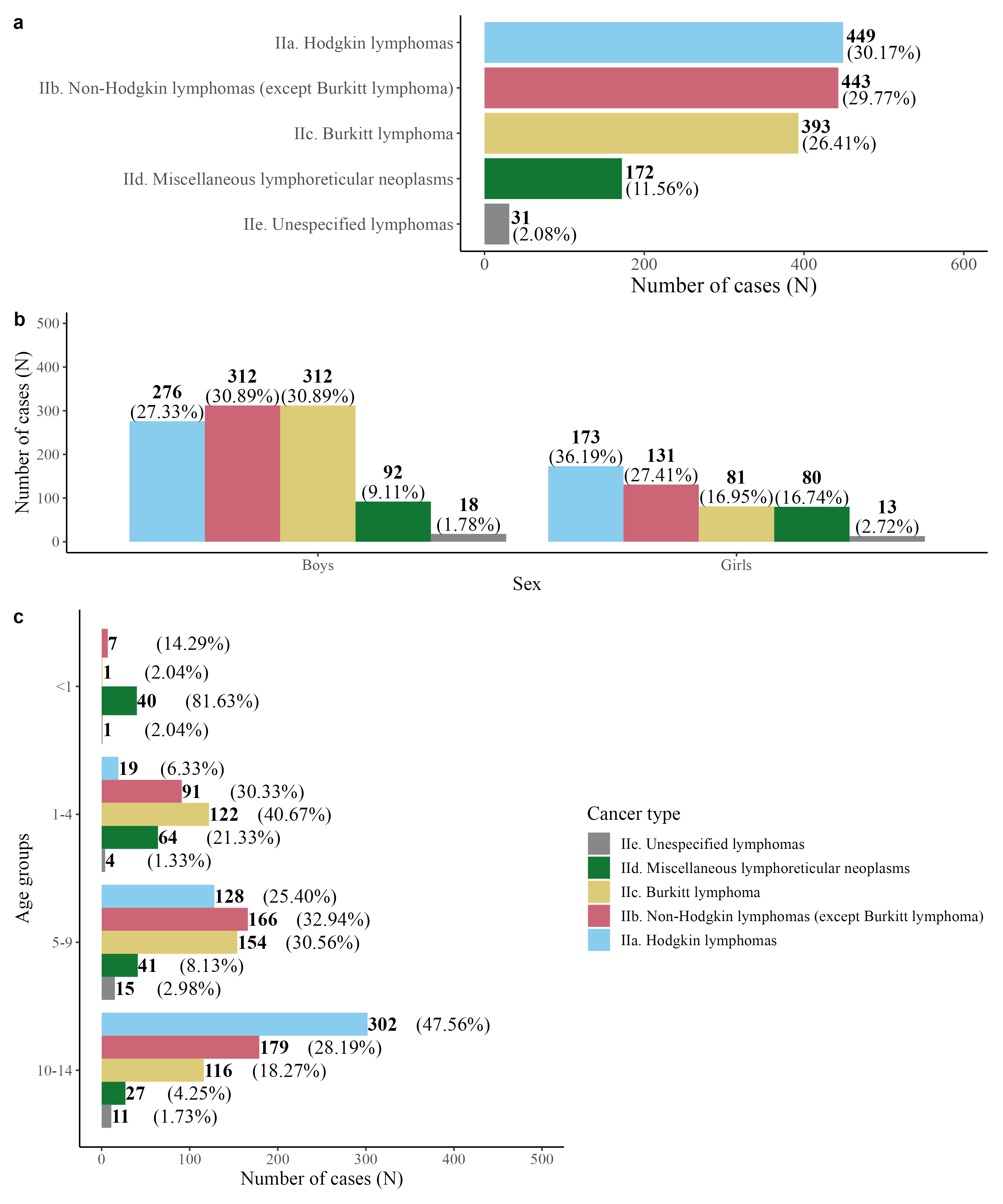


Figure S2: Number of cases and percentage of a) lymphoma subgroups, b) lymphoma subgroups by sex and c) lymphoma subgroups by age group.

Table S2: Age-standardised rates by the world population WHO (2000 - 2025) of all the leukaemias and leukaemias subgroups by sex and age group.

| **Sex** | **N** | **ASR** | **ASR 95% CI** |
| --- | --- | --- | --- |
| **Leukaemias, myeloproliferative diseases and myelodysplastic diseases** | | | |
| Both | 3,259 | 45.17 | (43.62;46.72) |
| Boys | 1,813 | 48.76 | (46.51;51.01) |
| Girls | 1,446 | 41.36 | (39.22;43.5) |
| **Ia. Lymphoid leukaemias** | | | |
| Both | 2,445 | 33.98 | (32.63;35.33) |
| Boys | 1,368 | 36.90 | (34.94;38.86) |
| Girls | 1,077 | 30.89 | (29.05;32.73) |
| **Ib. Acute myeloid leukaemias** | | | |
| Both | 561 | 7.70 | (7.05;8.35) |
| Boys | 306 | 8.17 | (7.25;9.09) |
| Girls | 255 | 7.21 | (6.33;8.09) |
| **Ic. Chronic myeloproliferative diseases** | | | |
| Both | 82 | 1.11 | (0.87;1.35) |
| Boys | 49 | 1.27 | (0.92;1.62) |
| Girls | 33 | 0.94 | (0.63;1.25) |
| **Id. Myelodysplastic syndrome** | | | |
| Both | 63 | 0.89 | (0.67;1.11) |
| Boys | 34 | 0.93 | (0.62;1.24) |
| Girls | 29 | 0.85 | (0.54;1.16) |
| **Ie. Unspecified and other specified leukaemias** | | | |
| Both | 108 | 1.48 | (1.21;1.75) |
| Boys | 56 | 1.48 | (1.09;1.87) |
| Girls | 52 | 1.48 | (1.07;1.89) |

Table S3: Age-standardised rates by the world population WHO (2000 - 2025) of all lymphomas and lymphomas subgroups by sex and age group.

| **Sex** | **N** | **ASR** | **ASR 95% CI** |
| --- | --- | --- | --- |
| **Lymphomas and reticuloendothelial neoplasms** | | | |
| Both | 1,488 | 19.84 | (18.84;20.84) |
| Boys | 1,010 | 26.23 | (24.6;27.86) |
| Girls | 478 | 13.08 | (11.9;14.26) |
| **IIa. Hodgkin lymphomas** | | | |
| Both | 449 | 5.77 | (5.24;6.3) |
| Boys | 276 | 6.95 | (6.13;7.77) |
| Girls | 173 | 4.52 | (3.85;5.19) |
| **IIb. Non-Hodgkin lymphomas (except Burkitt lymphoma)** | | | |
| Both | 443 | 5.90 | (5.35;6.45) |
| Boys | 312 | 8.08 | (7.18;8.98) |
| Girls | 131 | 3.60 | (2.99;4.21) |
| **IIc. Burkitt lymphoma** | | | |
| Both | 393 | 5.32 | (4.79;5.85) |
| Boys | 312 | 8.23 | (7.31;9.15) |
| Girls | 81 | 2.24 | (1.75;2.73) |
| **IId. Miscellaneous lymphoreticular neoplasms** | | | |
| Both | 172 | 2.43 | (2.06;2.8) |
| Boys | 92 | 2.51 | (2;3.02) |
| Girls | 80 | 2.35 | (1.84;2.86) |
| **IIe. Unspecified lymphomas** | | | |
| Both | 31 | 0.41 | (0.27;0.55) |
| Boys | 18 | 0.46 | (0.24;0.68) |
| Girls | 13 | 0.36 | (0.16;0.56) |

Table S4: Age-standardised rate by the European population from 2013 of all haematological neoplasms, leukaemias and lymphomas by sex and time periods.

| **Cancer type** | **Sex** | **1983 - 1994** | | **1995 - 2006** | | **2007 - 2018** | |
| --- | --- | --- | --- | --- | --- | --- | --- |
|  |  | **N** | **ASR (95%CI)** | **N** | **ASR (95%CI)** | **N** | **ASR (95%CI)** |
| **HN** | Both | 1,398 | 59.9  (56.8;63.1) | 1,686 | 65.6  (62.5;68.8) | 1,663 | 67.0  (63.8;70.2) |
|  | Boys | 835 | 69.47  (64.77;74.17) | 988 | 75.1  (70.4;79.6) | 1,000 | 78.3  (73.4;83.1) |
|  | Girls | 563 | 49.9  (45.7;54.0) | 698 | 55.7  (51.5;59.8) | 663 | 55.0  (50.9;59.2) |
| **Leukaemia** | Both | 974 | 42.3  (39.6;45.0) | 1,153 | 44.9  (42.3;47.5) | 1132 | 45.0  (42.4;47.7) |
|  | Boys | 540 | 45.5  (41.7;49.4) | 631 | 47.9  (44.2;51.7) | 642 | 49.6  (45.7;53.4) |
|  | Girls | 434 | 38.9  (35.2;42.6) | 522 | 41.7  (38.1;45.2) | 490 | 40.2  (36.6;43.8) |
| **Lymphoma** | Both | 424 | 17.6  (15.9;19.3) | 533 | 20.8  (19.0;22.5) | 531 | 22.0  (20.1;23.8) |
|  | Boys | 295 | 23.9  (21.2;26.7) | 357 | 27.1  (24.3;30.0) | 358 | 28.7  (25.7;31.7) |
|  | Girls | 129 | 11.0  (9.1;12.9) | 176 | 14.0  (11.9;16.1) | 173 | 14.9  (12.6;17.1) |

Table S5: Annual percentage change, 95% confidence intervals and p-values of all haematological neoplasms, leukaemias and lymphomas by sex and age groups during 1985-2016 in Spain.

| **Cancer type** | **Condition** | **N** | **1^st^ segment trend** | | | **2^nd^ segment trend** | | |
| --- | --- | --- | --- | --- | --- | --- | --- | --- |
|  |  |  | **Period** | **APC (95%CI)** | **p-value** | **Period** | **APC (95%CI)** | **p-value** |
| **HN** | Overall | 4,276 | 1985-1987 | 19.2  (6.3;32.1) | 0.004 | 1987-2016 | 0.2  (-0.2;0.6) | 0.33 |
|  | Boys | 2,548 | 1985-1988 | 14.5  (-1.2;30.2) | 0.070 | 1988-2015 | 0.2  (-0.3;0.7) | 0.433 |
|  | Girls | 1,820 | 1985-2016 | 0.7  (0.0;1.3) | 0.035 | - | - | - |
|  | <1 year | 234 | 1985-2016 | 0.5  (-1.6;2.5) | 0.633 | - | - | - |
|  | 1-4 years | 1,549 | 1985-1989 | 13.0  (-0.3;26.3) | 0.055 | 1989-2016 | -0.4  (-1.1;0.3) | 0.263 |
|  | 5-9 years | 1,297 | 1985-2016 | 0.8  (0.2;1.4) | 0.009 | - | - | - |
|  | 10-14 years | 1,196 | 1985-1993 | 5.9  (0.5;11.4) | 0.034 | 1993-2016 | 0.1  (-1.0;1.1) | 0.852 |
| **Leukaemia** | Overall | 2,930 | 1985-1988 | 15.3  (5.9;24.7) | 0.001 | 1988-2016 | 0.0  (-0.5;0.5) | 1.000 |
|  | Boys | 1,635 | 1985-1988 | 17.0  (-3.2;37.3) | 0.100 | 1988-2016 | 0.1  (-0.5;0.7) | 0.744 |
|  | Girls | 1,295 | 1985-2016 | 0.3  (-0.3;1.0) | 0.366 | - | - | - |
|  | <1 year | 191 | 1985-2016 | 0.5  (-1.6;2.5) | 0.633 | - | - | - |
|  | 1-4 years | 1,281 | 1985-1989 | 13.6  (-1.6;28.8) | 0.079 | 1989-2016 | -0.3  (-1.1;0.5) | 0.462 |
|  | 5-9 years | 843 | 1985-2016 | 0.5  (-0.1;1.2) | 0.132 | - | - | - |
|  | 10-14 years | 615 | 1985-2016 | 0.5  (-0.4;1.4) | 0.276 | - | - | - |
| **Lymphoma** | Overall | 1,346 | 1985-2016 | 1.0  (0.4;1.6) | 0.001 | - | - | - |
|  | Boys | 913 | 1985-2016 | 0.7  (0.0;1.4) | 0.050 | - | - | - |
|  | Girls | 433 | 1985-2016 | 1.6  (0.6;2.6) | 0.002 | - | - | - |
|  | <1 year | 43 | - | - | - | - | - | - |
|  | 1-4 years | 268 | 1985-2016 | -0.6  (-2.0;0.7) | 0.384 | - | - | - |
|  | 5-9 years | 454 | 1985-2016 | 1.3  (0.1;2.4) | 0.027 | - | - | - |
|  | 10-14 years | 581 | 1985-1994 | 10.6  (4.2;17.1) | 0.001 | 1994-2016 | -0.2  (-1.8;1.4) | 0.806 |
